# Prefrontal High Gamma in ECoG Tags Periodicity of Musical Rhythms in Perception and Imagination

**DOI:** 10.1523/ENEURO.0413-19.2020

**Published:** 2020-07-31

**Authors:** S.A. Herff, C. Herff, A.J. Milne, G.D. Johnson, J.J. Shih, D.J. Krusienski

**Affiliations:** 1 Digital and Cognitive Musicology Lab, École polytechnique fédérale de Lausanne, Lausanne, Vaud, 1015, Switzerland; 2 MARCS Institute for Brain, Behaviour and Development, Western Sydney University, Milperra, New South Wales, 2214, Australia; 3 School for Mental Health and Neuroscience, Faculty of Health, Medicine and Life Sciences, Maastricht University, Maastricht, Limburg, 6229, Netherlands; 4 Biomedical Engineering Program, Old Dominion University, Norfolk, Virginia, VA 23529, United States; 5 Department of Neurology, San Diego Health, University of California, San Diego, California, CA 92121, United States; 6 Advanced Signal Processing in Engineering and Neuroscience Lab, Virginia Commonwealth University, Richmond, Virginia, VA 23219, United States

**Keywords:** ECoG, high gamma, imagination, music perception, periodicity, rhythm

## Abstract

Rhythmic auditory stimuli are known to elicit matching activity patterns in neural populations. Furthermore, recent research has established the particular importance of high-gamma brain activity in auditory processing by showing its involvement in auditory phrase segmentation and envelope tracking. Here, we use electrocorticographic (ECoG) recordings from eight human listeners to see whether periodicities in high-gamma activity track the periodicities in the envelope of musical rhythms during rhythm perception and imagination. Rhythm imagination was elicited by instructing participants to imagine the rhythm to continue during pauses of several repetitions. To identify electrodes whose periodicities in high-gamma activity track the periodicities in the musical rhythms, we compute the *correlation between the autocorrelations* (ACCs) of both the musical rhythms and the neural signals. A condition in which participants listened to white noise was used to establish a baseline. High-gamma autocorrelations in auditory areas in the superior temporal gyrus and in frontal areas on both hemispheres significantly matched the autocorrelations of the musical rhythms. Overall, numerous significant electrodes are observed on the right hemisphere. Of particular interest is a large cluster of electrodes in the right prefrontal cortex that is active during both rhythm perception and imagination. This indicates conscious processing of the rhythms’ structure as opposed to mere auditory phenomena. The autocorrelation approach clearly highlights that high-gamma activity measured from cortical electrodes tracks both attended and imagined rhythms.

## Significance Statement

The possibility to capture high-frequency brain activity, such as high gamma, with high spatial and temporal resolution makes invasive brain recordings extremely valuable. We present new data from an invasive electrocorticographic (ECoG) study with a comparably large sample size. Deploying a new periodicity-tagging technique that extends the common frequency tagging, we found that high gamma in auditory areas tracks periodicity. Furthermore, we use the periodic nature of musical-stimuli as a neural footprint and found that high-gamma activity in the prefrontal cortex tracks periodicities of musical rhythms both during listening and imagination. The neural mechanisms involved in imagination in particular are ill understood. The present study provides evidence that the prefrontal cortex tracks periodicities in auditory stimuli during perception and imagination, and highlights the usefulness of musical stimuli for studying neural processes.

## Introduction

Neural populations match their activity patterns in response to repetitive, rhythmic auditory stimuli (Nozaradan et al., 2011, 2012, 2015; Nozaradan, 2014). However, the neural response to rhythmical stimuli is not exclusively driven by exogenous stimulus properties, such as an auditory stimulus, but is also shaped by endogenous top-down mechanisms, such as attention and imagination (Nozaradan et al., 2011). This suggests that neural activity in the context of repetitive auditory stimuli is not only worth investigating as a reactive process, triggered by external stimulation, but may also shed light on complex cognitive functions like imagination. The present analysis aims to further characterize neural activity in auditory perception and imagination, specifically in high-gamma activity.

### High Gamma

Recent medical advances have allowed music perception research investigating neural responses to auditory rhythms to venture beyond non-invasive EEG methodologies to invasive measurements such as the use of intracranial electrodes in epilepsy patients. In an intracranial study, Nozaradan et al. (2017) showed that a 0- to 30 Hz as well as a 30- to 100-Hz power band tracks the envelope of musical rhythms. In the present study, we aim to further explore the involvement of a different power band; high gamma.

Activity in the high-gamma band is much more localized (Miller et al., 2007) and thought to resemble ensemble spiking (Ray et al., 2008). Because of the small size of the generator area, frequencies above 70 Hz become increasingly unreliable to measure, let alone localize, using EEG. In electrocorticography (ECoG), the electrodes are deployed directly on the cortex rather than on the scalp. This enables accurate characterization of high gamma (or broadband gamma, ∼70–170 Hz). This is important, as high gamma can be linked to auditory attention, auditory perception, and appears to mark auditory segment boundaries (Leuthardt et al., 2011; Pei et al., 2011; Schalk and Leuthardt, 2011; Potes et al., 2012; Sturm et al., 2014; for review, see Cervenka et al., 2011). High-gamma activity can be used to decode speech from the brain (Pasley et al., 2012; C. Herff et al., 2015, 2019; Angrick et al., 2019a,b; Anumanchipalli et al., 2019). When listening to music, high gamma averaged across listeners correlates with the sound envelope of a musical piece in a data set with seven participants (Potes et al., 2012). Using the same data set with an additional three participants, Sturm et al. (2014) found a correlation between high gamma and the music envelope in four out of 10 participants. A recent study also suggests that high-gamma activity is not only involved in music listening but also music imagination (Ding et al., 2019). In this study, participants were asked to imagine the continuation of familiar musical pieces. High-gamma activity significantly exceeded the baseline that was measured before stimulus onset. Using lagged correlations between high gamma and the music’s envelope, the authors investigated the time course of the activation of different brain regions.

In the present study, we aim to further investigate the potential involvement of high gamma in music perception. However, rather than exploring familiar musical pieces, we focus on high gamma’s involvement in musical rhythm perception as well as imagination. Here, we are less concerned with the time courses of different brain regions’ activations, but rather aim to explore areas that capture the underlying periodicities of the rhythmic signal.

### Periodicity tagging

In the present study, we use autocorrelation representations of musical rhythms and high-gamma brain activity. This approach focuses on capturing and comparing the periodicities observed in the autocorrelation of the musical rhythm with those observed in high-gamma activity. This approach is inspired by, and related to, the widely used frequency-tagging approach; however, instead of comparing frequency components in the rhythmic envelope with frequency components in neural responses, it compares their periodicities (Henry et al., 2017; Rajendran et al., 2017; Lenc et al., 2018, 2019; Novembre and Iannetti, 2018; Nozaradan et al., 2018; Rajendran and Schnupp, 2019). For example, a rhythm might have many interonset intervals (where the onsets are not necessarily consecutive) of 500 ms and only a few such interonset intervals of 250 ms. Neural responses stimulated by such rhythms might, or might not, exhibit similar temporal periodicities. Because an autocorrelation captures the distribution of such periodicities in a signal, measuring the correlation between the autocorrelation of a rhythmic envelope and the autocorrelation of a neural response allows us to quantify how similar their periodicity distributions are. The correlation between these two autocorrelations is abbreviated here with ACC. Autocorrelations are invariant to phase, therefore they are not affected if there is a temporal delay between the two signals. Furthermore, there are a variety of different envelopes that can produce equivalent autocorrelations: we see this as an advantage because it is agnostic to the precise mechanism by which the periodicity is “coded” by the neural envelope. Indeed, there are various ways in which high-gamma activity could code the stimulus, not only through envelope matching, so a many-to-one matching is necessary when looking for areas of interest that track periodicity of stimuli. Here, we argue that if a high-gamma brain activity pattern represents or tracks the underlying periodicity of an acoustic signal, then it is most likely related to the stimulus. In summary, we specifically investigate here whether high-gamma activity during listening, as well as imagination of repetitive auditory rhythms captures the rhythms’ periodicities using a periodicity tagging approach.

## Materials and Methods

### Participants

ECoG data were recorded from eight patients (three female, five male, 22–42 years old) with pharmacoresistant epilepsy undergoing localization of epileptogenic zones and eloquent cortex before surgical resection. When questioned, no patients reported hearing deficits or any form of musical training. In all cases, a tumor was not the source for the seizures and no lesions were indicated by any electrode used for analysis. Patients participating in this study gave written informed consent and the study protocol was approved by the institutional review boards of Old Dominion University and Mayo Clinic, Florida. Patients were implanted with subdural electrode grids or strips based purely on their clinical need. Electrode locations were verified by co-registering preoperative MRI and postoperative computerized tomography scans. For combined visualization, electrode locations were projected to common Talairach space. There can be a small degree of positional error when projecting the individual co-registered electrodes onto the generic brain model for aggregation across participants. Electrode locations and activations were rendered using NeuralAct (Kubanek and Schalk, 2015). We recorded ECoG activity during rhythm perception and imagination of a total of 437 (151 left hemisphere, 286 right) subdural electrodes.

**Figure 2.**
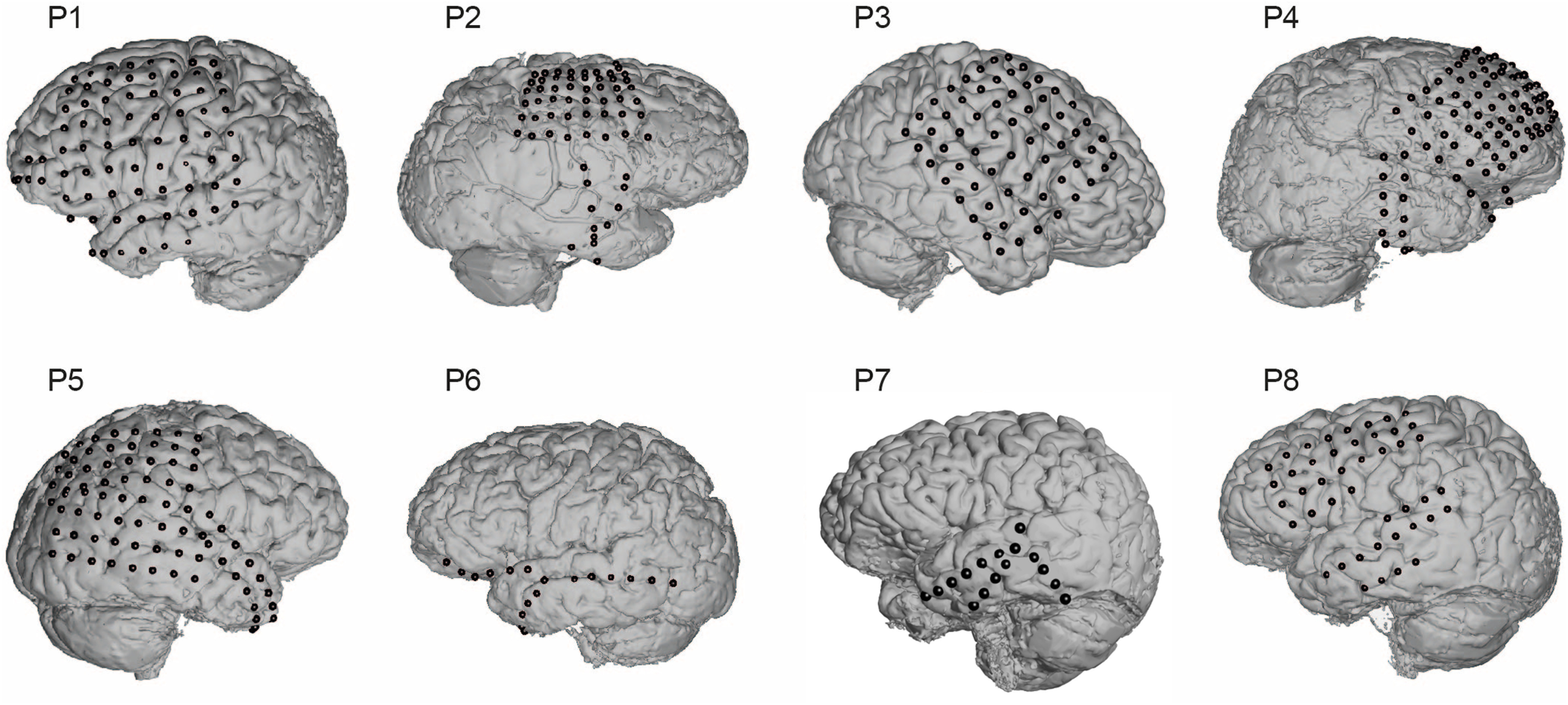
Electrode grid locations for all eight participants.

### Stimuli

**Figure 1.**
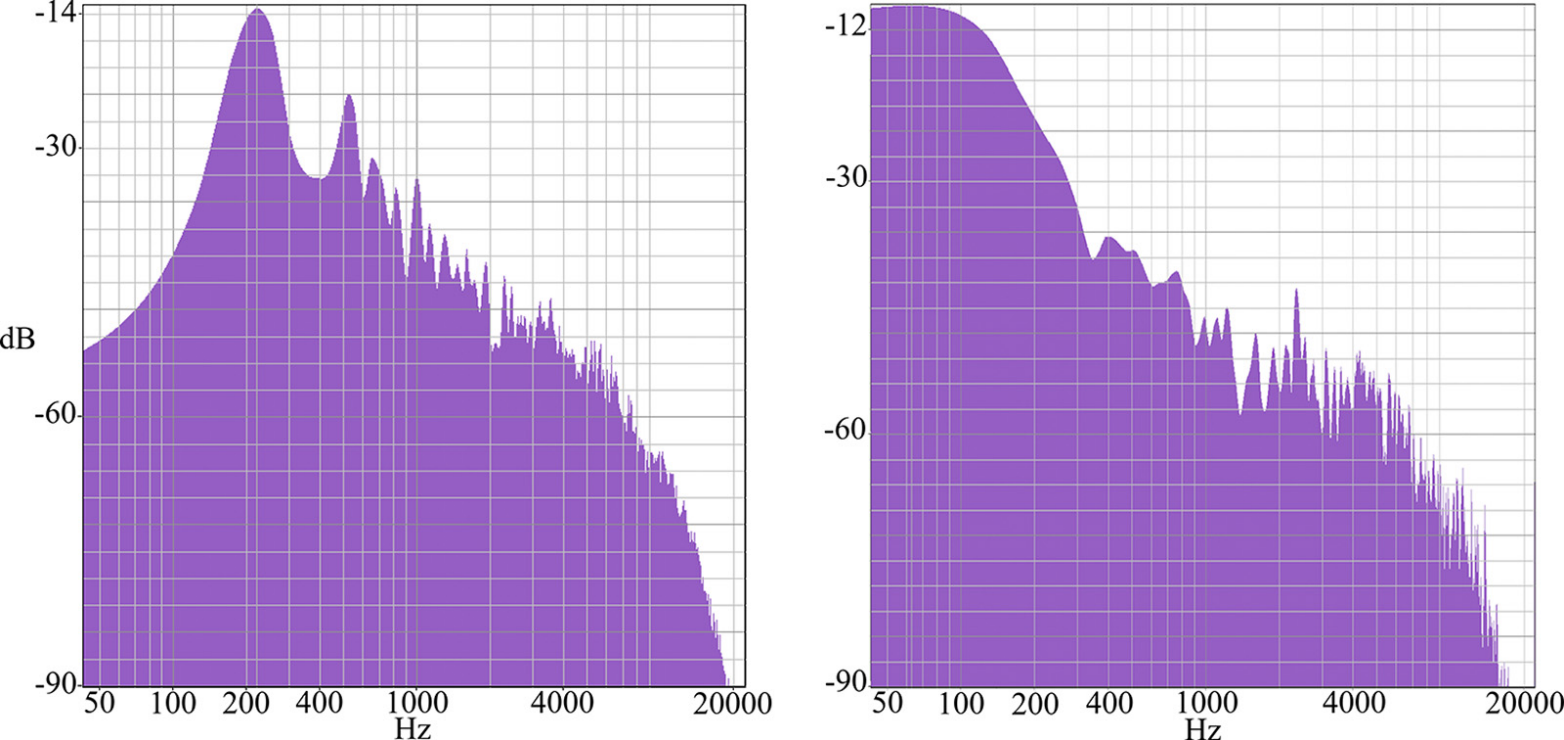
Spectra of the kick (right) and snare (left) sound.

The majority of research investigating neural activity to auditory rhythm stimuli use either complex speech or simple clicks, white noise, pure tones, or sine tones (Nozaradan et al., 2017). To increase ecologically validity for musical stimuli, we use kick-snare drum patterns. Both the kick and the snare sound showed no spectral peaks in the critical band (70–170 Hz). The kick’s fundamental spectral peak was at 63 Hz, and the snare peaked at 217 Hz. However, as naturalistic sounds were used, there was some energy present within the critical band. Figure 1 shows the spectra of the kick and the snare sound. Here, we analyze data of participants listening to two different musical rhythms. Each rhythm consists of eight pulses and four sounded events. The rhythms are being presented at either 120 or 140 bpm. Table 1 presents a summary of all rhythms. Rhythm 2 is a syncopated rhythm, that is, listeners will perceive a downbeat on the fifth element, despite there being no sounded event. We included a syncopated rhythm, as syncopation is typically considered to increase rhythmic complexity (Fitch and Rosenfeld, 2007); this allows us to explore periodicity tagging in a more complex rhythm. Furthermore, a control was implemented by a condition that presented white noise instead of a rhythm.

**Table 1:** Overview of the musical rhythms

Rhythm	Sounded events	Sequence							
Unsyncopated	4	K	x	S	x	K	x	S	x
Syncopated	4	K	x	S	K	x	S	x	x

K represents a sounded kick; S represent a sounded snare, and x represent a non-sounded element.

### Procedure

Each participant passively listened to each rhythm in condition-blocks of six repetitions in 120 bpm (12 s) and eight repetitions in 140 bpm (13.7 s). After each rhythm block, the rhythm dropped out (i.e., became silent) for two repetitions in the 120-bpm condition (4 s) and two repetitions during the 140-bpm condition (3.4 s), and participants were instructed to imagine the rhythm to continue (imagining condition). After the imagining condition, the rhythms became audible for another two repetitions in both tempo conditions. Each block appeared twice throughout the experiment. The order of rhythm blocks was randomized. For the listening and imagining blocks, participants were instructed not to tap along with the rhythm or to move, and adherence to these instructions was confirmed for each participant through investigator observation. For each rhythm block, additional trials were performed that required the participant to tap the events of the rhythms using their dominant hand. These intermingled tapping trials as well as additional rhythm blocks using different rhythms, were not included in the present analysis. ECoG signals were simultaneously recorded throughout the experiment.

### ECoG data collection

Data from the electrode grids or strips (Ad-Tech Medical Instrument Corporation, 1-cm spacing) were bandpass filtered between 0.5 and 500 Hz and recorded using g.USB amplifiers (g.tec medical engineering) at a sampling rate of 1200 Hz. Data recording and stimulus presentation were facilitated by BCI2000 (Schalk et al., 2004). Electrode grids for all eight participants can be seen in Figure 2.

### Data analysis

**Figure 3.**
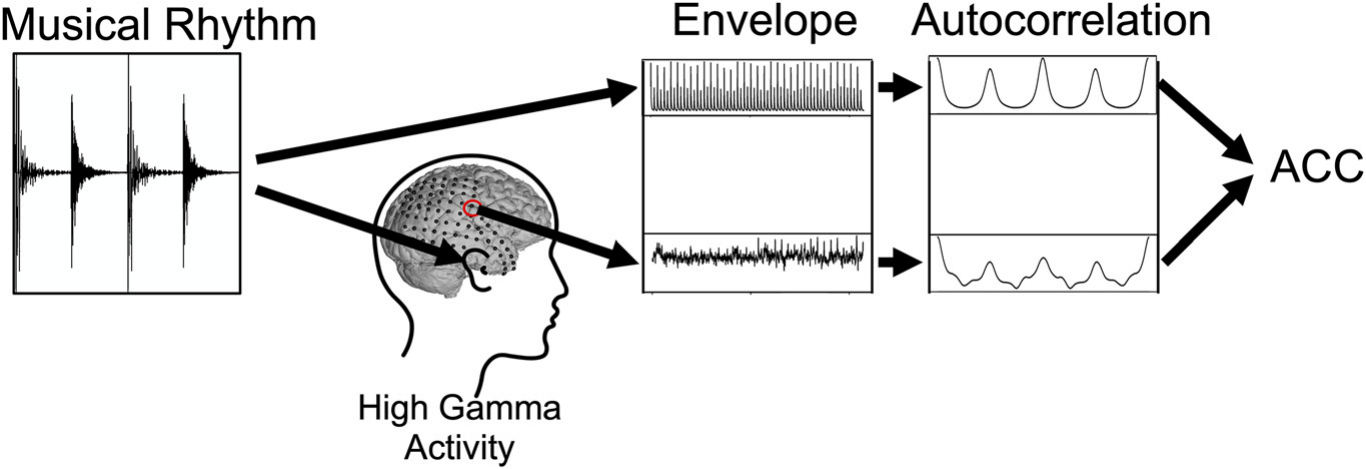
Schematic representation of the data analysis. The left most panel depicts the original waveform of a musical rhythm. The rhythm in this example is a “K x S x K x S x,” with K being the kick, S the snare, and x a pause. First, we extracted the envelope of the continuously looped presentation of the rhythm, as shown in the middle top panel. The top right panel shows the autocorrelation of the rhythm’s envelope. Note that the shown autocorrelation vector corresponds to the length of the original rhythm to emphasize the relationship between waveform and autocorrelation. For the actual analysis, we used the whole autocorrelation vector over all repeated presentations of the rhythm up to the Nyquist frequency. Simultaneously, we measured high-gamma activity from cortex electrodes while participants are listening (or imagining) the rhythm. Similar to the musical rhythm, we extracted the envelope of the high-gamma activity and calculated the autocorrelation. In a last step, we correlated the autocorrelation of high-gamma envelope and musical rhythm envelope to obtain our dependent variable: ACC.

Separately for each individual participant, electrode, tempo (120 vs 140 bpm), audio condition (listening vs imagine), and rhythm (unsyncopated: K x S x K x S x vs syncopated: K x S K x S x x), we extracted the absolute Hilbert envelope of high-gamma activity. We used elliptic Infinite impulse response (IIR) low-pass and high-pass filters to bandpass filter the ECoG signals between 70 and 170 Hz and applied an elliptic IIR notch filter to attenuate the first harmonic of the 60-Hz line noise. The Hilbert transform was then used to extract the envelope. We calculated the circular autocorrelation over all repeated presentations of the rhythm up to the Nyquist frequency. This was done by taking the real component of an inverse DFT of a pointwise multiplication of a DFT of the high-gamma time series and its complex conjugate and then dividing each element by the maximum element of the vector. The same transformation was conducted on the envelope of the musical rhythm’s waveform. High gamma and musical rhythm autocorrelation were correlated with one another to obtain the ACC. The resulting ACC between high-gamma brain activity and musical rhythms were used to statistically assess whether high gamma tracks musical rhythms. This process is schematically represented in Figure 3. Visually, ACC can be described as the correlation between the top and bottom right panels in Figure 3. As a control, we extracted high-gamma activity, envelope, and autocorrelation also for a condition where participants were listening to white noise instead of the actual musical rhythms and calculated ACC.

We deployed a Bayesian mixed effect model to predict the *correlation between the autocorrelation* of high gamma and musical rhythms (ACC, scaled to mean* *=* *0, SD* *=* *1) based on *rhythm* (unsyncopated vs syncopated), and *audio condition* (listen vs imagine), and *signal* (white noise vs rhythm). The model was provided with a random effect for *participant*, electrode, tempo (120 vs 140 bpm), and *presentation* (first vs second time a condition was shown), resulting in the maximal random effect structure as justified by the experimental design (Barr et al., 2013). The models were implemented in the R-environment (R-Core-Team, 2013) using the brms-package (Bürkner, 2017, 2018). The signal coefficient in combination with its interaction terms allows us to inspect the evidence in favor of whether high-gamma activity meaningfully tracks musical rhythms, while controlling for brain activity that a participant, at a given electrode location, would show when listening to a length-matched white noise segment instead of the actual musical rhythm. In other words, our model is provided with the information of how high the ACCs value between high gamma and musical rhythms can be expected to be for every individual electrode and participant, simply because of an auditory stimulus (here, we use white noise as a control). The model predicts the difference to this baseline, when participants are actually listening or imagining the rhythms. The model was provided with a weakly informative prior Student’s *t*_(3,0,1)_ and ran on four chains with 1000 warm-ups and 10,000 iterations each.

## Results

In a first step, we explore whether high-gamma tracking periodicities of the musical rhythms can be observed on a broad spatial scale. For this, we deployed Bayesian mixed effects models that compare ACCs obtained when participants listened or imagined the rhythms to ACCs obtained from the baseline. The baseline is the ACC between a musical rhythm and high gamma of a given participant and electrode when listening to white noise. Table 2 shows coefficient estimates (β), 95% confidence intervals (CIs), as well as evidence ratios for the hypothesis that there is elevated brain-wide high gamma tracks the musical rhythm. For convenience, we denote with * conditions that show “significant” tracking of periodicities at an α = 0.05 level (evidence ratios* *>* *19; see Milne and Herff, 2020). The results of Table 2 are derived by performing the hypothesis tests shown in Table 3 on the fitted model shown in Table 4.

**Table 2:** Summary of evidence observed in each condition whether broad spatial high gamma tracks the periodicities of musical rhythms more than baseline

Rhythm	Audio condition	β	95% CI_β_	Evidence ratio
Unsyncopated	Listen	0.1136	0.0827 to 0.1445	>9999^*^
Unsyncopated	Imagine	–0.0435	–0.0749 to –0.0125	0.0114
Syncopated	Listen	0.0741	0.0432 to 0.1052	>9999^*^
Syncopated	Imagine	0.1368	0.1049 to 0.1678	>9999^*^

We obtain strong evidence for broad spatial high-gamma tracking of the envelope of musical rhythms in the syncopated rhythms during listening and imagining, as well as in the unsyncopated rhythm during listening. However, we do not observe evidence for whole-brain tracking of the unsyncopated rhythm during imagination.

* effects that can be considered significant at a α = 0.05 level.

**Table 3:** Hypotheses performed on the model shown in **Table 4**

Rhythm	Audio condition	Hypothesis test
Unsyncopated	Listen	Rhythm > 0
Unsyncopated	Imagine	Rhythm + Rhythm:Imagined > 0
Syncopated	Listen	Rhythm + Rhythm:Syncopated > 0
Syncopated	Imagine	Rhythm + Rhythm:Syncopated + Rhythm:Imagined + Rhythm:Syncopated:Imagined > 0

The reference was placed at the white noise condition, unsyncopated, listening condition. Rhythm indicates that the actual rhythm rather than white noise was heard. Unsyncopated and syncopated refer to the two different rhythms used. Imagined indicates that the rhythms were not played, and instead participants were asked to imagine them.

**Table 4:** Model summary

	Scaled ACC	
Predictors	Estimates	CI (95%)
Intercept	–0.67	–0.73 to –0.61
Rhythm	0.11	0.08 to 0.15
Syncopated	0.44	0.40 to 0.48
Imagined	0.82	0.78 to 0.85
Rhythm.Syncopated	–0.04	–0.09 to 0.01
Rhythm.Imagined	–0.16	–0.21 to –0.11
Syncopated.Imagined	–0.00	–0.05 to 0.05
Rhythm.Syncopated.Imagined	0.22	0.15 to 0.29
*N*_Electrode_	518	
Observations	16576	
Marginal *R*^2^/conditional *R*^2^	0.216 / 0.629	
σ^2^	0.41	

On a broad spatial scale, we observe strong evidence (all evidence ratios* *>* *9999) in favor of high-gamma autocorrelations tracking the autocorrelations of the musical rhythms in the syncopated rhythm during listening and imagination, and in the unsyncopated rhythm during listening, but not imagination. When comparing the two rhythms, the unsyncopated and the syncopated rhythms show comparable ACCs in the listening condition (β = 0.04, EE_β_ =* *0.03, 95% CI_β_ = –0.004 to 0.83, evidence ratio = 13.46). In the imagination condition, however, we obtain strong evidence for higher ACCs in the syncopated rhythm compared with the unsyncopated condition (β = 0.18, EE_β_* *=* *0.03, 95% CI_β_ = 0.14 to 0.22, evidence ratio = >9999*). Although we do not observe tracking on a broad spatial scale in the unsyncopated imagination condition, this does not imply that there are no electrodes for which the high-gamma activity tracks the musical rhythms, as can be seen in the electrode-wise results.

**Figure 4.**
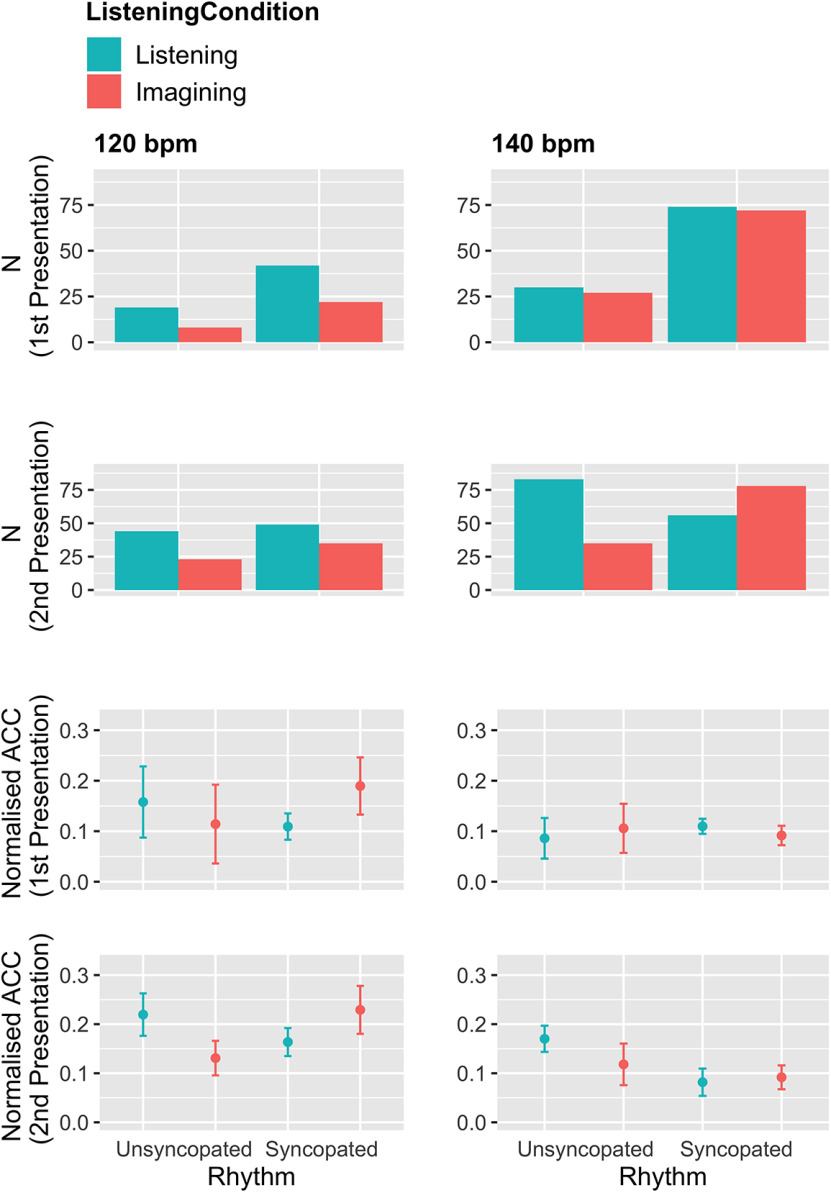
Number (first and second row) and magnitudes (third and fourth row) of electrodes that significantly track musical rhythms in their high-gamma activity, pooled across participants and electrodes. Significance was defined by exceeding participant-wise 99% of the ACCs between musical rhythms and high gamma during the white-noise control condition. All conditions contain electrodes that significantly track the musical rhythms. Normalized ACC values were obtained by subtracting the significance thresholds from the observed ACCs. Error bars represent 95% CIs.

Figure 4 shows counts of the electrodes that significantly track the musical rhythms’ periodicities, as well as their normalized ACC. We calculated significance thresholds for each participant and rhythm individually. For this, we used the distribution of correlations between the ACC of a musical rhythm and the ACC of high-gamma activity while listening to length-matched white noise segments. Correlations that exceed 99% of this distribution are deemed significant. Normalized ACC values were obtained by subtracting the ACC when listening to length-matched white noise instead of listening or imagining the musical rhythms. Each electrode in a given participant was normalized by the white noise ACC of the same electrode in that participant. As can be seen in Figure 4, each condition contains electrodes in which high-gamma autocorrelations track the autocorrelations of the respective musical rhythms.

Figure 4 also suggests an increase in the number of significant electrodes between first and second presentation of each condition (i.e., higher bars in the second row compared with the first). A Bayesian mixed model supports this. The model predicts normalized ACC based on *presentation number* (first vs second), while controlling for *participant* and *electrode*. The model reveals an increase in normalized ACC in all conditions (all evidence ratios* *>* *65*). This can be seen in Table 5.

**Table 5:** Summary of evidence observed that normalized ACCs are higher during the second presentation compared with the first presentation of each condition

Rhythm	Tempo	Audio condition	β	95% CI_β_	Evidence ratio
Unsyncopated	120	Listen	0.24	0.15 to 0.34	>9999^*^
Unsyncopated	120	Listen	0.20	0.11 to 0.30	>9999^*^
Unsyncopated	140	Imagine	0.32	0.23 to 0.42	>9999^*^
Unsyncopated	140	Imagine	0.28	0.18 to 0.37	>9999^*^
Syncopated	120	Listen	0.17	0.08 to 0.27	799^*^
Syncopated	120	Listen	0.12	0.03 to 0.22	65.56^*^
Syncopated	140	Imagine	0.14	0.05 to 0.24	136.93^*^
Syncopated	140	Imagine	0.21	0.06 to 0.30	3999^*^

We obtain strong evidence that normalized ACCs are higher in the second compared with the first presentation in all conditions.

* effects that can be considered significant at an α = 0.05 level.

To investigate the potential overlap between significant electrodes in listening and imagination we used a Bayesian mixed effects models predicting *SignificanceDuringImagination* (binary factor with 1 = significant, 0 = not significant), based on *SignificanceDuringListening* (and vice versa), while controlling for *rhythm, tempo, participant, presentation*, and *electrode*. We observe very strong evidence that *SignificanceDuringListening* predicts *SignificanceDuring Imagination* (β = 1.83, EE_β_* *=* *0.25, 95% CI_β_ = 1.43–2.24, evidence ratio = >9999*) and vice versa (β = 2.51, EE_β_* *=* *0.26, 95% CI_β_ = 2.01–2.94, evidence ratio = >9999*). This suggests high predictive information between the electrodes that are significant in listening and those that are significant during imagination. Further insight is provided in the topography section of the results.

**Figure 5.**
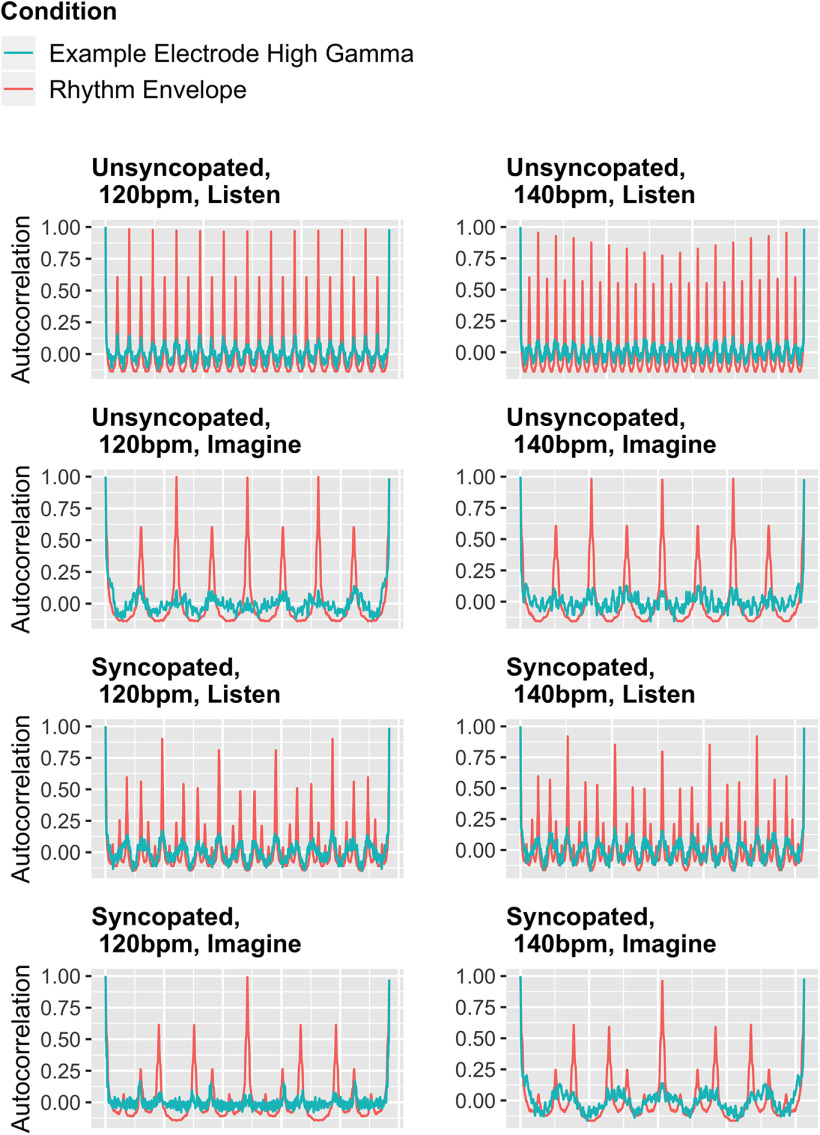
Autocorrelations of the musical rhythm conditions and prefrontal example electrodes (blue-yellow, prefrontal cluster in Fig. 6). The *x*-axis represents the sample (time). The autocorrelations of the listen condition look different to the autocorrelations of the imagine condition, because there were more repetitions, thus samples, in the listen condition (six repetition at 120 bpm over 12 s; eight repetitions at 140 bpm over 13.7 s) before the audio dropped out, than there were samples in the silent imagine condition (two repetitions in both tempi; 4 s at 120 bpm, 3.4 s at 140 bpm). There are electrodes in which high-gamma autocorrelations (blue) significantly track the musical rhythms autocorrelations (red) in all conditions.

To visualize the tracking, Figure 5 shows examples for each condition. The red line shows the autocorrelation of a given musical rhythm. The blue line shows the autocorrelation of an example electrode.

This study is predominantly concerned with high gamma; however, we performed the same analysis on the beta band (12–30 Hz) to see whether high-gamma activity carries information that is not contained in other frequency bands. We chose beta because it was suggested by the reviewers and prior work suggests an involvement of beta in neural processing of musical rhythms (Chang et al., 2016). We observed strong evidence that there are more electrodes that significantly correlate with the musical rhythms’ autocorrelations using high gamma compared with beta (β = 2.17, EE_β_* *=* *0.72, 95% CI_β_ = 0.98–3.4, evidence ratio* *=* *2799*). Furthermore, the increase in normalized ACC between first and second presentation that is observed in all conditions in high gamma is not observed in beta in any condition (all evidence ratios < 5.78), with the exception of the unsycopated rhythm at 140 bpm in the imagined condition (evidence ratio* *=* *799*). However, it is worth mentioning that we also found some electrodes that correlated with the musical rhythms’ autocorrelations in the beta autocorrelations.

### Topography

**Figure 6.**
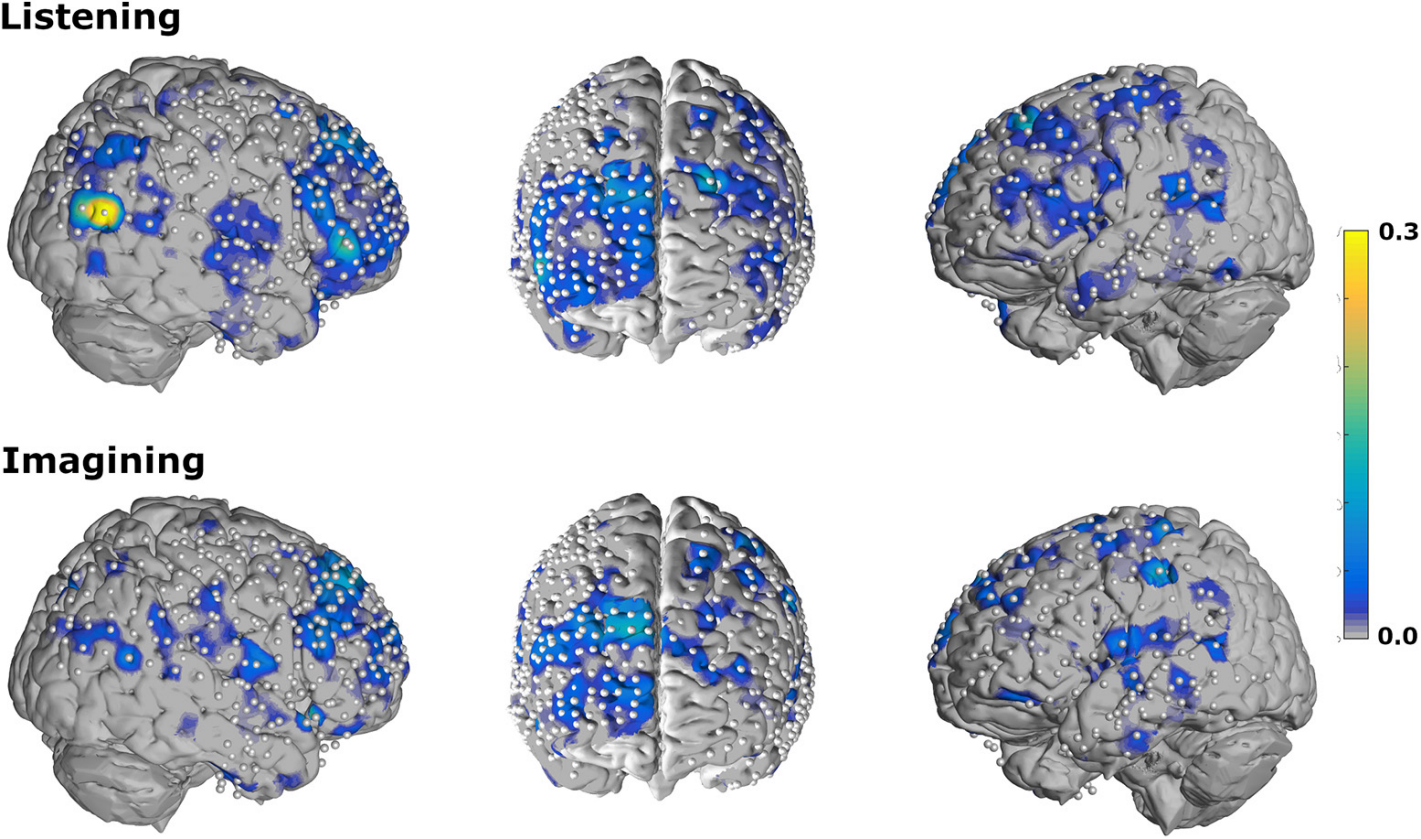
A joint brain map for all participants across all conditions. Heat maps visualize mean normalized ACC across all rhythms and tempo. Significant ACCs can be observed particularly in the frontal areas of the right hemisphere. These ACCs are also significant during the imagine condition.

To localize the effect, we plotted all electrodes on a joint brain map. Figure 6 shows heat maps of mean normalized ACC for listening (Fig. 6, top) and imagining (Fig. 6, bottom) across all rhythms and tempi.

## Discussion

The present study investigated the involvement of high gamma in listening as well as imagining musical rhythms using brain activity of eight participants measured through invasive ECoG. Bayesian mixed effects models provided compelling support that high-gamma activity tracks the envelope of musical rhythms. Specifically, we deployed an analytical approach that emphasizes the periodicity in musical rhythms by investigating correlations between the autocorrelations of musical rhythms and the autocorrelations of high-gamma brain activity. In all listening conditions the models support the conclusion that high-gamma activity captures the periodicity in musical rhythms. We observe the same in all but one condition: when participants are imagining the rhythms, rather than listening to them. Taken together, it appears that during imagination, neural populations display similar high-gamma activity that tracks the envelope of the imagined stimulus, usually observed when acoustic stimuli are actually present. This may be preliminary support for the notion that, on a neural level, imagination involves activity of the reactive neural response associated with the presence of the stimulus.

The present finding supports previous ECoG studies that highlight the importance of high-gamma activity in auditory processing (Leuthardt et al., 2011; Pei et al., 2011; Schalk and Leuthardt, 2011; Pasley et al., 2012; Potes et al., 2012; Sturm et al., 2014; Herff et al., 2015; see Cervenka et al., 2011). Specifically, our results replicate the findings that high gamma tracks music envelopes (Sturm et al., 2014). Such replications are important, because ECoG studies operate with very small sample sizes. Furthermore, we extend the finding to imagination, and a periodicity tagging approach. The direct approach of directly correlating high gamma with stimulus envelope deployed by (Sturm et al., 2014) relies on relatively long segments, clean data, and a phase locking. Furthermore, correlating high gamma with the stimulus envelope can only identify neural population that engage in envelope matching. Yet, there are various ways in which high-gamma activity could theoretically code the stimulus. The present approach is able to identify neural populations that engage in envelope matching as well as those that match any form of distinct activity pattern to the periodicities of the stimuli. As such, we put the present approach forward as a useful tool to identify brain regions of interest. The identified regions could then be further analyzed to characterize the nature of the activity pattern that tracks the periodicities of the stimuli. It is important to note that the present approach correlates the two autocorrelations with one another. It is possible that other metrics of similarity, such as cosine similarity, Weissman score, or shared mass, could work equally well or even better. Future work could investigate the benefits of more sophisticated similarity measures.

The unsyncopated and the syncopated rhythms show comparable ACCs in the listening condition. This is worth noting as the syncopated rhythm could be considered the more complicated rhythm (Fitch and Rosenfeld, 2007). The stronger tagging of the syncopated rhythm compared with the unsyncopated rhythm in the imagination condition is unexpected. A possible explanation could be, that the syncopated rhythm may be more interesting and engaging for participants, having a “groove” that makes it easier to entrain. A different explanation considering the order in which the conditions were presented is provided in the limitations section.

High-gamma activity showed a greater number of significant electrodes compared with beta activity. High gamma also shows a strong increase in normalized ACCs between first and second presentation in all conditions. This increase was only seen in one condition for beta (unsyncopated, 140 bpm, imagined). The strong evidence for an increase in normalized ACCs between first and second presentation in high gamma, but not beta, may suggest that some form of higher order auditory processing is involvement in periodicity tagging in high gamma that improves with increased exposure. A possible candidate could be a prediction-based mechanism that shows clearer activation patterns when familiar with a rhythm. While high gamma showed more significant electrodes, there were some electrodes that also showed significant tagging of the musical rhythms’ periodicities in the beta band. This is interesting because beta can be reliable captured in EEG, whereas high gamma cannot. A future study could investigate whether periodicity tagging can be shown using EEG and the ACCs in the beta band.

### Topography

Electrodes with significant ACCs can be found in auditory areas in the superior temporal gyrus and in frontal areas on both hemispheres. Numerous significant electrodes are observed on the right hemisphere which is in accordance with previous findings (Thaut et al., 2014). However, due to the better coverage of the right hemisphere compared with the left hemisphere, we cannot draw conclusions about hemispherical dominance (151 left hemisphere, 286 right hemisphere). Of particular interest is the large cluster of electrodes in the right prefrontal cortex that are active during both rhythm perception and imagined perception, which indicate conscious processing of the rhythm structure as opposed to mere auditory phenomena. This finding mirrors research that also observed frontal high gamma when imagining familiar music (Ding et al., 2019). The previous study also found elevated high-gamma activation in the temporal lobe during imagination. Here, we did not observe that high gamma in the temporal lobe represents the periodicities of the musical rhythms during imagination like the prefrontal cortex does. However, this could simply be due to a difference in methodology. The previous study (Ding et al., 2019) focused on areas that show elevated high-gamma activity and/or areas where gamma activity tracks the music’s envelope. The present study uses musical rhythms rather than familiar music, and focuses on areas that track the rhythms’ periodicities, regardless of overall activity. However, any area that closely tracks the audio envelope in the present dataset would have been identified by our periodicity-tagging approach, thus further research is required to elucidate the role of the temporal lobe during imagination.

### Limitations

An important limitation in the present design is that what we and others (Ding et al., 2019) liberally term “imagination” is in fact an “imaginary continuation” of the rhythms. In theory, such a continuation could be functionally distinct from unprompted imagination. In fact, it is possible that if the imagination condition would have lasted longer, then potentially the high-gamma representation of the rhythms’ periodicity may have diverged. This is an empirical question for a future study. Despite using stimuli that showed no spectral peaks in the critical band (70–170 Hz), when using naturalistic drum sounds, it is impossible to avoid energy across the spectrum. It is therefore possible that that the neural patterns observed are event related potentials, rather than ongoing neural activity. However, the prefrontal location, as well as activity during imagination would require further thought to explain through event related potentials. Furthermore, the unsyncopated imagination condition that did not show brain-wide significant tracking of the rhythms periodicity in high gamma urges caution interpretation of the present results. This is, because the condition was the simpler rhythm. If anything, we would have expected this condition to show the strongest effect. A possible explanation lies in the fact that this condition was always tested first. Potentially, participants were not yet familiar with the imagination task to evoke a reliable effect. Some support for this explanation can be gained from the increase in normalized ACC as well as number of significant electrodes between first and second presentation of the conditions. Furthermore, as common in invasive brain studies, we were operating with small participant numbers, and despite our best efforts of making the most of the data at hand, by deploying a Bayesian framework, we simply may not have the statistical power to compensate for all sources of random variability.

## Conclusion

Deploying an analytical approach that emphasizes the periodicity in musical rhythms, we found that high-gamma brain activity in auditory areas tracks periodicity when listening to musical rhythms. Furthermore, we found that high-gamma activity in the prefrontal cortex tracks periodicity of musical rhythms both during listening and imagination.

## Synthesis

Reviewing Editor: Satu Palva, University of Helsinki Decisions are customarily a result of the Reviewing Editor and the peer reviewers coming together and discussing their recommendations until a consensus is reached. When revisions are invited, a fact-based synthesis statement explaining their decision and outlining what is needed to prepare a revision will be listed below. The following reviewer(s) agreed to reveal their identity: Gabriele Arnulfo, Sylvie Nozaradan.

Synthesis

Stimuli. The authors used kick-snare drum sounds. Please provide some details on the spectral content on these sounds. That is, does it contain acoustic energy in low portions of the spectrum, namely overlapping with the 70-170 Hz band investigated in the study? In which case, it would be worth mentioning, as tracking of the stimuli in the listening condition could be simply due to event-related responses rather than ongoing oscillatory activity.

Procedure. The authors have rephrased the section describing the trials, with tapping trials intermingled in the experiment. However, it would be worth discussing this explicitly somewhere in the Discussion as well. That is, tapping the rhythm is likely to impact on the way the same rhythm is further imagined in a session. Relatedly, was tapping on the syncopated rhythm significantly different than on the unsyncopated rhythm (as we could expect, given the difference in complexity between the two rhythms)? If so, it could explain the significant difference in imagining the syncopated vs. unsyncopated rhythm, and this is not an anecdotal result.

Strategies for keeping pace could be different from subjects to subjects and might include mental counting and tapping. The authors included tapping blocks in their study design. Why are not these data included in the current study? A significant difference between muscular activated (due to rhythmic tapping) and purely auditory electrodes in terms of ACC results should more strongly prove their results.

ECoG data collection. It is now mentioned in this section that there were more electrodes located on the right vs. left hemisphere. Please also add this information explicitly in the Discussion when discussing the more pronounced tracking of the stimuli in the right vs. left hemisphere.

Discussion. The first paragraph mentions 9 patients instead of 8. Please correct according to the Methods section. Caption of Table 2. There is a confusion between syncopated and unsyncopated rhythm. It is said that there was strong evidence for significant tracking in the unsyncopated rhythm condition imagination. Also, there is an incomplete sentence in the caption.

Discussion. Third paragraph. It is said: “The stronger tagging of the unsycnopated rhythm compared to the syncopated rhythm in the imagination condition is surprising”. Please correct according to the Methods, where the opposite is described, that is, stronger tagging when imagining the syncopated vs. unsyncopated rhythm. First, in the introduction when presenting the autocorrelation approach (page 6 - Periodicity tagging paragraph) the sentence it self is vague. Please consider rephrasing.

Figure 4. Please explicitly mention the exact location of the example electrode used to illustrate tracking. Moreover, it is not clear why the AC signal showed for the rhythm envelope is not identical between listen and imagine conditions for each rhythm and tempo. Please clarify. Figure doesn't have a proper x-axis.

Fig.5 some electrodes seem off specifically those at the anterior temporal lobe

## References

[B1] Angrick M, Herff C, Johnson GD, Shih J, Krusienski DJ, Schultz T (2019a) Interpretation of convolutional neural networks for speech spectrogram regression from intracranial recordings. Neurocomputing 342:145–151. 10.1016/j.neucom.2018.10.080

[B2] Angrick M, Herff C, Mugler E, Tate MC, Slutzky MW, Krusienski DJ, Schultz T (2019b) Speech synthesis from ECoG using densely connected 3D convolutional neural networks. J Neural Eng 16:036019 10.1088/1741-2552/ab0c59 30831567 PMC6822609

[B3] Anumanchipalli GK, Chartier J, Chang EF (2019) Speech synthesis from neural decoding of spoken sentences. Nature 568:493–498. 10.1038/s41586-019-1119-1 31019317 PMC9714519

[B4] Barr DJ, Levy R, Scheepers C, Tily HJ (2013) Random effects structure for confirmatory hypothesis testing: keep it maximal. J Mem Lang 68:255–278. 10.1016/j.jml.2012.11.001 PMC3881361 24403724

[B5] Bürkner P (2017) brms: an R package for Bayesian multilevel models using Stan. J Stat Soft. Advance online publication. Retrieved August 29, 2017. doi: 10.18637/jss.v080.i01 10.18637/jss.v080.i01

[B6] Bürkner P (2018) Advanced Bayesian multilevel modeling with the R package brms. arXiv 1705.11123

[B7] Cervenka MC, Nagle S, Boatman-Reich D (2011) Cortical high-gamma responses in auditory processing. Am J Audiol 20:171–180. 10.1044/1059-0889(2011/10-0036) 22158634 PMC3848128

[B8] Chang A, Bosnyak DJ, Trainor LJ (2016) Unpredicted pitch modulates beta oscillatory power during rhythmic entrainment to a tone sequence. Front Psychol 7:327. 10.3389/fpsyg.2016.00327 27014138 PMC4782565

[B9] Ding Y, Zhang Y, Zhou W, Ling Z, Huang J, Hong B, Wang X (2019) Neural correlates of music listening and recall in the human brain. J Neurosci 1468–1418. 10.1523/JNEUROSCI.1468-18.2019 PMC6786812 31501297

[B10] Fitch WT, Rosenfeld AJ (2007) Perception and production of syncopated rhythms. Music Percept 25:43–58. 10.1525/mp.2007.25.1.43

[B11] Henry MJ, Herrmann B, Grahn JA (2017) What can we learn about beat perception by comparing brain signals and stimulus envelopes? PLoS One 12:e0172454. 10.1371/journal.pone.0172454 28225796 PMC5321456

[B12] Herff C, Heger D, de Pesters A, Telaar D, Brunner P, Schalk G, Schultz T (2015) Brain-to-text: decoding spoken phrases from phone representations in the brain. Front Neurosci 9:217. 10.3389/fnins.2015.00217 26124702 PMC4464168

[B13] Herff C, Diener L, Angrick M, Mugler E, Tate MC, Goldrick MA, Krusienski DJ, Slutzky MW, Schultz T (2019) Generating natural, intelligible speech from brain activity in motor, premotor and inferior frontal cortices. Front Neurosci 13:1267. 10.3389/fnins.2019.01267 31824257 PMC6882773

[B14] Kubanek J, Schalk G (2015) NeuralAct: a tool to visualize electrocortical (ECoG) activity on a three-dimensional model of the cortex. Neuroinformatics 13:167–174. 10.1007/s12021-014-9252-3 25381641 PMC5580037

[B15] Lenc T, Keller PE, Varlet M, Nozaradan S (2018) Neural tracking of the musical beat is enhanced by low-frequency sounds. Proc Natl Acad Sci USA 115:8221–8226. 10.1073/pnas.1801421115 30037989 PMC6094140

[B16] Lenc T, Keller PE, Varlet M, Nozaradan S (2019) Reply to Rajendran and Schnupp: frequency tagging is sensitive to the temporal structure of signals. Proc Natl Acad Sci USA 116:2781–2782. 10.1073/pnas.1820941116 30696761 PMC6386670

[B17] Leuthardt EC, Gaona C, Sharma M, Szrama N, Roland J, Freudenberg Z, Solis J, Breshears J, Schalk G (2011) Using the electrocorticographic speech network to control a brain–computer interface in humans. J Neural Eng 8:e036004. 10.1088/1741-2560/8/3/036004 21471638 PMC3701859

[B18] Miller KJ, Leuthardt EC, Schalk G, Rao RPN, Anderson NR, Moran DW, Miller JW, Ojemann JG (2007) Spectral changes in cortical surface potentials during motor movement. J Neurosci 27:2424–2432. 10.1523/JNEUROSCI.3886-06.2007 17329441 PMC6673496

[B19] Milne AJ, Herff SA (2020) The perceptual relevance of balance, evenness, and entropy in musical rhythms. Cognition. doi: 10.1016/j.cognition.2020.104233. 10.1016/j.cognition.2020.104233 32629203

[B20] Novembre G, Iannetti GD (2018) Tagging the musical beat: neural entrainment or event-related potentials? Proc Natl Acad Sci USA 115:E11002–E11003. 10.1073/pnas.1815311115 30425178 PMC6255208

[B21] Nozaradan S (2014) Exploring how musical rhythm entrains brain activity with electroencephalogram frequency-tagging. Philos Trans R Soc Lond B Biol Sci 369:20130393. 25385771 10.1098/rstb.2013.0393 PMC4240960

[B22] Nozaradan S, Peretz I, Missal M, Mouraux A (2011) Tagging the neuronal entrainment to beat and meter. J Neurosci 31:10234–10240. 10.1523/JNEUROSCI.0411-11.2011 21753000 PMC6623069

[B23] Nozaradan S, Peretz I, Mouraux A (2012) Selective neuronal entrainment to the beat and meter embedded in a musical rhythm. J Neurosci 32:17572–17581. 10.1523/JNEUROSCI.3203-12.2012 23223281 PMC6621650

[B24] Nozaradan S, Zerouali Y, Peretz I, Mouraux A (2015) Capturing with EEG the neural entrainment and coupling underlying sensorimotor synchronization to the beat. Cereb Cortex 25:736–747. 10.1093/cercor/bht261 24108804

[B25] Nozaradan S, Mouraux A, Jonas J, Colnat-Coulbois S, Rossion B, Maillard L (2017) Intracerebral evidence of rhythm transform in the human auditory cortex. Brain Struct Funct 222:2389–2404. 10.1007/s00429-016-1348-0 27990557

[B26] Nozaradan S, Keller PE, Rossion B, Mouraux A (2018) EEG frequency-tagging and input–output comparison in rhythm perception. Brain Topogr 31:153–158. 10.1007/s10548-017-0605-8 29127530

[B27] Pasley BN, David SV, Mesgarani N, Flinker A, Shamma SA, Crone NE, Knight RT, Chang EF (2012) Reconstructing speech from human auditory cortex. PLoS Biol 10:e1001251. 10.1371/journal.pbio.1001251 22303281 PMC3269422

[B28] Pei X, Leuthardt EC, Gaona CM, Brunner P, Wolpaw JR, Schalk G (2011) Spatiotemporal dynamics of electrocorticographic high gamma activity during overt and covert word repetition. Neuroimage 54:2960–2972. 10.1016/j.neuroimage.2010.10.029 21029784 PMC3020260

[B29] Potes C, Gunduz A, Brunner P, Schalk G (2012) Dynamics of electrocorticographic (ECoG) activity in human temporal and frontal cortical areas during music listening. Neuroimage 61:841–848. 22537600 10.1016/j.neuroimage.2012.04.022 PMC3376242

[B30] Rajendran VG, Schnupp JWH (2019) Frequency tagging cannot measure neural tracking of beat or meter. Proc Natl Acad Sci USA 116:2779–2780. 10.1073/pnas.1820020116 30696762 PMC6386709

[B31] Rajendran VG, Harper NS, Garcia-Lazaro JA, Lesica NA, Schnupp JWH (2017) Midbrain adaptation may set the stage for the perception of musical beat. Proc R Soc B 284:20171455 10.1098/rspb.2017.1455 PMC5698641 29118141

[B32] Ray S, Crone NE, Niebur E, Franaszczuk PJ, Hsiao SS (2008) Neural correlates of high-gamma oscillations (60–200 Hz) in macaque local field potentials and their potential implications in electrocorticography. J Neurosci 28:11526–11536. 10.1523/JNEUROSCI.2848-08.2008 18987189 PMC2715840

[B33] R-Core-Team (2013) R: a language and enviornment for statistical computing. Vienna: R Foundation for Statistical Computing.

[B34] Schalk G, Leuthardt EC (2011) Brain-computer interfaces using electrocorticographic signals. IEEE Rev Biomed Eng 4:140–154. 10.1109/RBME.2011.2172408 22273796

[B35] Schalk G, McFarland DJ, Hinterberger T, Birbaumer N, Wolpaw JR (2004) BCI2000: a general-purpose brain-computer interface (BCI) system. IEEE Trans Biomed Eng 51:1034–1043. 10.1109/TBME.2004.827072 15188875

[B36] Sturm I, Blankertz B, Potes C, Schalk G, Curio G (2014) ECoG high gamma activity reveals distinct cortical representations of lyrics passages, harmonic and timbre-related changes in a rock song. Front Hum Neurosci 8:798. 10.3389/fnhum.2014.00798 25352799 PMC4195312

[B37] Thaut M, Trimarchi P, Parsons L (2014) Human brain basis of musical rhythm perception: common and distinct neural substrates for meter, tempo, and pattern. Brain Sci 4:428–452. 10.3390/brainsci4020428 24961770 PMC4101486

